# Towards European harmonisation of healthcare for patients with rare immune disorders: outcome from the ERN RITA registries survey

**DOI:** 10.1186/s13023-020-1308-x

**Published:** 2020-01-30

**Authors:** Riccardo Papa, Andrew Cant, Christoph Klein, Mark A. Little, Nico M. Wulffraat, Marco Gattorno, Nicolino Ruperto, on behalf of ERN RITA Council

**Affiliations:** 1 Clinica Pediatrica e Reumatologia, IRCCS Istituto Giannina Gaslini, Genoa, Italy; 2 Great North Children’s Hospital & Institute for Cellular Medicine, University of Newcastle, Newcastle upon Tyne, UK; 3 Department of Pediatrics, Dr. von Hauner Children’s Hospital, University Hospital, LMU Munich, Munich, Germany; 4 Trinity Health Kidney Centre, Tallaght University Hospital, Dublin, Ireland; 5 Department of Pediatrics, Section Pediatric Rheumatology, Wilhelmina Children’s Hospital, University Medical Centrum Utrecht, Utrecht, Netherlands; 6 Centre for Autoinflammatory Diseases and Immunodeficiencies, IRCCS Istituto Giannina Gaslini, Genoa, Italy; 7 UOSID Centro Trial, PRINTO, IRCCS Istituto Giannina Gaslini, Genoa, Italy

**Keywords:** Primary immunodeficiency, Autoinflammatory diseases, Autoimmune disorders, European research network, Disease registry

## Abstract

The Rare Immunodeficiency, AutoInflammatory and AutoImmune Disease (RITA) network is a European Research Network (ERN) that brings together the leading centres for rare immune disorders. On April 2018 an online survey was sent to all RITA members in order to facilitate the harmonization of data collection in rare immune disorders registries. Currently, as many as 52 different registries collect data on rare immune disorders, of whom 30 (58%) are dedicated primarily to autoimmune diseases, 15 (29%) to primary immunodeficiencies and 12 (23%) to autoinflammatory disorders. Improving data on patient safety, outcome, and quality of life measures is warranted to unfold the full potential of RITA registries.

## Background

Immunology is a young discipline, originating from the discovery of innate and adaptive immune responses in the last century [1]. The field has evolved rapidly from the identification of primary immunodeficiencies (PID) predisposing to infection, to the recognition of specific rare immune disorders with disturbed immunoregulatory balances, called Autoinflammatory disorders (AID), and others with defective tolerance to self-antigens, also known as Autoimmune diseases (AI) [2–4]. Thus, errors in the immune system can result not only in specific clinical entities, but also in conditions with two or three features of these pathologic processes [5, 6].

A multidisciplinary care for patients with rare immune disorders (RID) using complex diagnostic evaluation and highly specialized therapies is nowadays required. For example, genome-wide analysis platforms and functional immune assays are rapidly developing diagnostic tests shared across all subthemes and usually not available in all centres [7, 8]. Polyvalent immunoglobulin therapy has revolutionised the outlook for antibody deficient patients, specific anti-cytokine (anti-TNF, anti-IL-1 etc) treatments have transformed the lives of patients with AID and AI, and stem cell and gene-based therapies, originally for PID, are now being applied for all the RID and for the first time enable patients to be completely cured with no need for ongoing medical care [9–11].

### RITA proposal

RITA is a European research network (ERN) that brings together the leading European centres with expertise in diagnosis and treatment of RID. ERNs are virtual networks involving healthcare providers (HCP) and family organization across Europe to facilitate discussion on complex or rare diseases that require highly specialised treatment, concentrating knowledge and resources (https://ec.europa.eu/health/ern_en) [12]. The foundation of RITA has been developed from the resources of successful, already existing, highly specialised international scientific societies, registries and websites, and in particular European Society for Immune Deficiencies (ESID) with ESID registry, Paediatric Rheumatology European Society (PRES), International Society for Systemic Auto-Inflammatory Diseases (ISSAID), Paediatric Rheumatology International Trials Organisation (PRINTO) with EUROFEVER and PHARMACHILD projects, European Vasculitis Society (EUVAS), and BEHCET International.

The main aims of RITA are: i) to provide state of the art for comprehensive clinical care for children and adults with RID, harmonizing diagnostic and therapeutic guidelines across Europe, thus ensuring for every patient an equal access to excellent expertise and care and reducing patient search of cross-border healthcare; ii) to establish sustainable alliances within European centres to accelerate diagnosis, improve access to treatment, and develop transitional care for patients with RID, maximizing the cost-effective use of resources and facilitating mobility of expertise; iii) to ensure the correct codification of quality control on diagnostic tests and targeted therapy, establishing a common tool for pharmacovigilance in these rare conditions; iv) to reinforce epidemiological surveillance and basic research on RID; v) to train future leaders in the field, securing their sustainability in an international perspective, and promote the awareness of RID between clinicians, carers, patients, family organizations and politicians, advocating better resources and measure to ensure early diagnosis by screening and enhanced symptom recognition.

Thus, providing a common shared medical platform for all affected patients and HCPs, independently of nationality and ethnicity, is a major attempt of the network [13, 14]. We report herein the results of an international survey within RITA members about all existing registries and research networks with the aim to harmonise and facilitate future data merging for research and clinical purposes.

## Methods

Each RITA member was invited with a formal email to participate in the present survey (Additional file 1). In brief, each member was asked about personal involvement in any registry projects and covered role (coordinator, participant or just knower). We collected data about registries name, website, boundaries extension (national or international with involved countries), considered group of diseases (PID, AID, and/or AI), number of enrolled patients, and types of collected data. Members shared registry protocol and/or case report form when possible.

All data were collected and analysed using the Excel program. Descriptive data were reported as absolute numbers and percentages for categorical variables.

## Survey results

The online survey was sent to all 126 RITA members during April 2018, of whom 45 are HCPs and 8 are patients and family organizations (Table 1). Informative responders were 75 (60%) because 15 members delegated the response to other members of the network. Collectively, 27 members are coordinating 25 registries; 53 members are participating in 38 registries, and 27 members knew the existence of 16 registries without participating. Only two members are not involved in any registries.

**Table 1:** Registries survey results overview

*Are you aware of any Registry of Diseases covered by the ERN RITA?*	Number (%)
Responder RITA members	90/126 (71)
Responder HCPs	39/45 (87)
Responder family organizations	4/8 (50)
Delegate to other RITA members	15/90 (17)
Countries	14
Informative responders	75/126 (60)
Coordinators	27/75 (36)
Participants	53/75 (71)
Knew registry without participating	27/75 (36)
Not knew any registry	2/75 (3)
Registries	52
*Is it a National or International Registry?*	
International	16/52 (31)
National	36/52 (69)
*Which area of diseases is covered by the Registry you are involved?*	
Autoimmunity	29/52 (56)
Primary immunodeficiency	15/52 (29)
Autoinflammation	20/52 (39)
Only autoinflammation	12/52 (23)
*What type of data are you collecting?*	
Demography	42/52 (81)
Diagnosis	45/52 (87)
Signs and symptoms	40/52 (77)
Therapy	39/52 (75)
Safety	28/52 (54)
Genetic	20/52 (39)
Laboratory	35/52 (67)
Imaging	5/52 (10)
Biobank	15/52 (29)

Data about 52 different registries were collected across 14 European countries (Table 2). Almost 50% of registries collect data on AI, while others are dedicated to PID or AID (respectively 15 registries, 29%, and 12 registries, 23%). Fifteen registries (29%) enrolled patients with a single specific disorder, in particular three registries for monogenic forms of systemic lupus erythematosus, two registries for Kawasaki disease or Behcet disease, and single registry for juvenile dermatomyositis, juvenile systemic sclerosis, juvenile idiopathic arthritis (JIA)-related uveitis, systemic JIA, Blau syndrome, sarcoidosis, Guillain-Barre syndrome, and myasthenia gravis.

**Table 2:** Registries among RITA network

Name	Web site	Since	Diseases	Countries	HCPs (number)	Enrolled patients (number)
AID-Registry	https://prst.gpoh.de/aid/	NR	AID	Germany	42	1376
ATTRA Clinical Registry	http://attra.registry.cz/	2002	AI	Czech Republic	42	NR
Czech National Guillain-Barrè Syndrome Registry	NR	NR	AI	Czech Republic	NR	NR
Banque nationale de données Maladies Rares (BNDMR)	http://www.bndmr.fr/	2013	AI, AID	France	950	NR
Blau cohort study	NR	NR	AID	NR	NR	NR
Brainworks: Childhood CNS Vasculitis and Inflammatory Brain Diseases	http://www.sickkids.ca/research/brainworks/	NR	AI, AID	NR	NR	NR
Centre des maladies rares (CEMARA)	www.cemara.org	NR	AI, AID	France	NR	NR
Centre de Référence Déficits Immunitaires Héréditaires (CEREDIH)	https://www.ceredih.fr/	NR	AID, PID	France	111	NR
Centre de Référence National Cytopénies auto-immunes de l’enfant (CEREVANCE)	http://www.cerevance.org/	NR	AI	France	NR	NR
CID and PIDs registry	https://old.iss.it/site/cnmr/registrare/cid/entry.aspx	NR	PID	Italy	NR	NR
Czech Registry of ANCA-associated vasculitis	Not available for public	NR	AI	Czech Republic	NR	1000
Dutch PID registry	None	NR	PID	Netherland	NR	NR
European Society for Blood and Marrow Transplantation (EBMT) registry	http://www.ebmt.org/ebmt-patient-registry	1974	PID, AI, AID	NR	NR	NR
European Society for Immunodeficiencies (ESID) registry	https://esid.org	2004	PID, AI, AID	NR	126	> 28,000
EULAR WEB Library	https://www.eular.org/eular_imaging_library_portal.cfm	NR	AI	NR	NR	NR
Eurofever	https://www.printo.it/eurofever	NR	AID	Argentina, Armenia, Australia, Austria, Belgium, Brazil, Canada, Chile, China, Croatia, Czech Republic, Denmark, France, Georgia, Germany, Greece, Hungary, India, Israel, Italy, Japan, Latvia, Lebanon, Lithuania, Netherlands, Oman, Poland, Romania, Russian Federation, Saudi Arabia, Serbia, Slovakia, Slovenia, Spain, Sweden, Switzerland, Turkey, United Kingdom, United States	NR	4057
French Vasculitis Study Group (FVSG) registry	https://www.vascularites.org/	NR	AI	France	NR	4000
Hemophagocytic lymphoistiocytosis (HLH) registry	https://histiocytesociety.org/main-website-pages/clinical-studies/hlh-registry	NR	PID	NR	NR	NR
IMMUNAID	Not yet launched	NR	AID	France, Germany, Italy, Netherlands, Spain, Turkey	NR	NR
Inception Cohort for juvenile Systemic sclerosis	http://www.juvenile-scleroderma.com/	2014	AI	Austria, Belgium, Czech Republic, Denmark, Germany, Italy, Latvia, Portugal, Russia, Slovenia, Spain, Sweden, Turkey, United Kingdom, United States of America, Argentine, Brazil, Israel, India	30	97
Infevers	https://infevers.umai-montpellier.fr/web/	2001	AID	NR	NR	1628
Innsbruck registry for Adamantiades-Behcet disease: Retrospective and prospective data collection	https://ctc.tirol-kliniken.at/page.cfm?vpath=oeffentliche&action=viewdetail&studie=7852	2009	AID	Austria	NR	NR
IPINET	www.aieop.org/web/operatori-sanitari/gruppi-di-lavoro/immunodeficienze/	1999	PID	Italy	62	3064
Irish Rare Kidney Disease registry and biobank	http://www.tcd.ie/medicine/thkc/research/rare.php/	2012	AI	Ireland	9	668
JIR cohort	http://www.fondationres.org/fr/jircohorte	NR	AI, AID	France, Switzerland, Belgium, Luxembourg, Morocco, Germany	69	4805
JULES: Registro nacional Lupus eritematoso sistémico	NR	NR	AI	Spain	45	4024
Kawanet	Not active	2011	AID	France	65	466
Kaw-race	http://www.imas12.es/redcap/	NR	AI	Spain	NR	NR
Kidbiosep Cohort	None	NR	AI	France	NR	324
MALATTIE RARE LAZIO V 1.3	https://www.regione.lazio.it/asponline/servizi/malattie_rare/login.php	NR	PID	Italy	NR	192
Myasthenia Gravis Registry (MYREG)	http://myreg.registry.cz		AI	Czech Republic	9	776
National Biobank	None	NR	AID	Italy	NR	2727
Orbis Maladies Rares	None	NR	AI	France	NR	NR
Paediatric MS database (OPTIMISE)	None	NR	AID	United Kingdom	NR	100
PCID registry	www.pcid-study.org	2011	PID	International	NR	NR
Paediatric Behçet’s Disease (PEDBD) registry	Not active	NR	AID	France, Italy, Morocco, Iran, Turkey, Germany	NR	233
PEDOLUP	http://www.fondationres.org/fr/jircohorte	NR	AI	France, Switzerland, Belgium, Luxembourg, Morocco, Germany	NR	90
Pharmachild	www.printo.it	NR	AI	NR	NR	10,100
PIDcare	pidcare.se	NR	PID	Sweden	25	2500
PID-NET	www.pid-net.org	2009	PID, AI, AID	Germany	NR	2600
Rare Disease Cohort (RADICO)-AcoStill	http://www.radico.fr/fr/connaitre-radico/nos-cohortes-et-autres-programmes-associes/80-radico/140-cohorte-radico-acostill	2014	AID	France	8	NR
Registro AIJ sistémica y enfermedad de Still del adulto	Not active	NR	AID	Spain	NR	NR
Registro Nazionale Malattie Rare (RNMR)	https://old.iss.it/site/cnmr/mrregi/mrlogin.asp	2001	PID, AI, AID	Italy	NR	NR
Registro Nacional Dermatomiositis Juvenil	None	NR	AI	Spain	NR	NR
Registro Lombardia Malattie Rare (ReLMaR)	http://malattierare.marionegri.it/content/view/91/99/	2015	PID, AI, AID	Italy	54	NR
Registro de Uvéitis Asociada a Artritis idiopatica juvenil	http://uveitis.pacifico-meetings.com/index.php	2013	AI	Spain	16	NR
Rational Use of Biologics in rare Refractory Immune mediated inflammatory diseases Consortium (RUBRIC)	https://www.rubricregister.nl/	NR	AI	Netherlands	NR	NR
Sarcoidosis UK	https://www.sarcoidosisuk.org/	NR	AI	United Kingdom	NR	NR
Stem Cell Transplant for primary Immune Deficiencies in Europe (SCETIDE)	www.scetide.org	NR	PID	NR	NR	NR
UK JSLE Cohort Study and Repository	https://www.liverpool.ac.uk/translational-medicine/research/ukjsle/about/	2006	AI	United Kingdom	23	500
UK and Ireland Vasculitis Rare Disease Group (UKIVAS) Registry	https://research.ndorms.ox.ac.uk/ukivas/	NR	AI	United Kingdom, Ireland	27	5000
UKPIN Registry	http://www.ukpin.org.uk/registry/ukpin-registry-login	NR	PID	United Kingdom	NR	4600

NR = not reported

More than 55,000 patients with RID are enrolled in a disease registry in Europe (Table 2). The majority of registries (36; 69%) enrols patients from national boundaries and only one registry collect data from two countries (UKIVAS registry). Among the international registries, five collect data on PID (ESID, EBMT, SCETIDE, PCID and HLH registry), four with AI (Pharmachild, BrainWorks, EULAR web library, and JIR cohort), and three are devoted to AID (Eurofever, Infevers, and ImmunAID). The ESID registry and JIR cohort also collect data on AID. Other international registries are devoted to a single specific disorder, i.e. monogenic forms of systemic lupus erythematosus, juvenile systemic sclerosis, Behcet disease and Blau syndrome.

Data usually collected in these registries are demography, diagnosis, clinical manifestations, laboratory tests and treatment, while genetic and imaging data are less frequently reported (respectively in 39 and 10% of registries). A treatment safety profile is reported in 29 registries (56%). Collectively, fifteen biobank are counted.

## Discussion

RITA intends to ensure that all patients with RID are included in Europe wide registries to facilitate harmonization of patient care, audit of compliance with agreed guidelines and to create a platform for research.

The present survey of registries clearly shows how the network needs a common plan for inventory the clinical data about patients with RID. Furthermore, key parameters of patient safety, including the use of medicines and medical technologies, communication issues and breaches in continuity of care, as well as outcome data (e.i. mortality, morbidity and disease complications) or quality of life measures are not usually reported in the current registries. Differences in the number of patients registered across countries may be related to a different capacity of each HCP to collect and enter the data in the exiting registries. RITA aims at supporting these HCPs, promoting registry-oriented researches and looking for funding to supports to registry users.

In this line, the European Commission Department for Health & Food Safety recently developed the European Rare Disease Registry Infrastructure (ERDRI) as part of the European Platform on Rare Diseases Registration (EU RD Platform, available at https://eu-rd-platform.jrc.ec.europa.eu). By promoting interoperability between data sources, the EU RD Platform helps reaching the necessary critical numbers to conduct studies and research on rare disease. The major objective of the project is to tackle the enormous fragmentation of patient data contained in hundreds of patient registries across Europe. The ERDRI strategy is to create an inventory of registries about rare diseases, in which 27 characteristics for each registry were considered, and a list of 16 minimum data element to be registered by all registries across Europe, providing instructions on how and in which format each data element should be registered. A phonetic hashing to prevent duplicate registration of patients has been proposed and freely available at https://eupid.eu/.

The next RITA aim will be to support the conversion of all existing registries about RID to the ERDRI format to ensure their interoperability with other European rare disease registries with the belief that cross fertilisation of ideas would enable these registries to be further developed than would be otherwise. In fact, disease registries play an important role in the development of orphan drugs, so essential to the treatment of patients with rare disorders. RITA will seek to facilitate collaboration with industry for the further development of such treatments. It is expected that other registries in the meanwhile identified (e.g. the Spanish Registry of Primary Immunodeficiencies - REDIP), will be identified and invited to participate to any future initiative.

## Conclusions

The survey highlighted the pivotal role of national and international organizations in Europe to collect and organize clinical data on immune diseases, allowing the rapidly growing knowledge on these rare disorders, creating research networks and providing significant numbers of data to support new discoveries in the field. RITA network could improve the coordination of these numerous entities, supporting initiatives of collaboration and cooperation. As a first attempt, the present survey revealed that the collection of key parameters about patient safety, as well as outcome data and quality of life measures should be improved among the registries of RITA network.

## Supplementary information

**Additional file 1.** The registries survey sheet.

## Data Availability

The datasets used and/or analysed during the current study are available from the corresponding author on reasonable request.

## Abbreviations

AI: Autoimmune diseases
AID: Autoinflammatory disorders
ERDRI: European Rare Disease Registry Infrastructure
ERN: European Reseach Network
ESID: European Society for Immune Deficiencies
EU RD Platform: European Platform on Rare Diseases Registration
EUVAS: European Vasculitis Society
HCP: Health Care Provider
ISSAID: International Society for Systemic Auto-Inflammatory Diseases
JIA: Juvenile Idiopathic Arthritis
PID: Primary immunodeficiencies
PRES: Paediatric Rheumatology European Society
PRINTO: Paediatric Rheumatology International Trials Organisation
REDIP: Spanish Registry of Primary Immunodeficiencies
RID: Rare immune disorders
