# Supplementary figures and images for: Towards European harmonisation of healthcare for patients with rare immune disorders: outcome from the ERN RITA registries survey
Source: Orphanet J Rare Dis. 2020 Jan 30;15:33. doi: 10.1186/s13023-020-1308-x (PMC6993334; doi:10.1186/s13023-020-1308-x)

**Additional file 1.** The registries survey sheet.


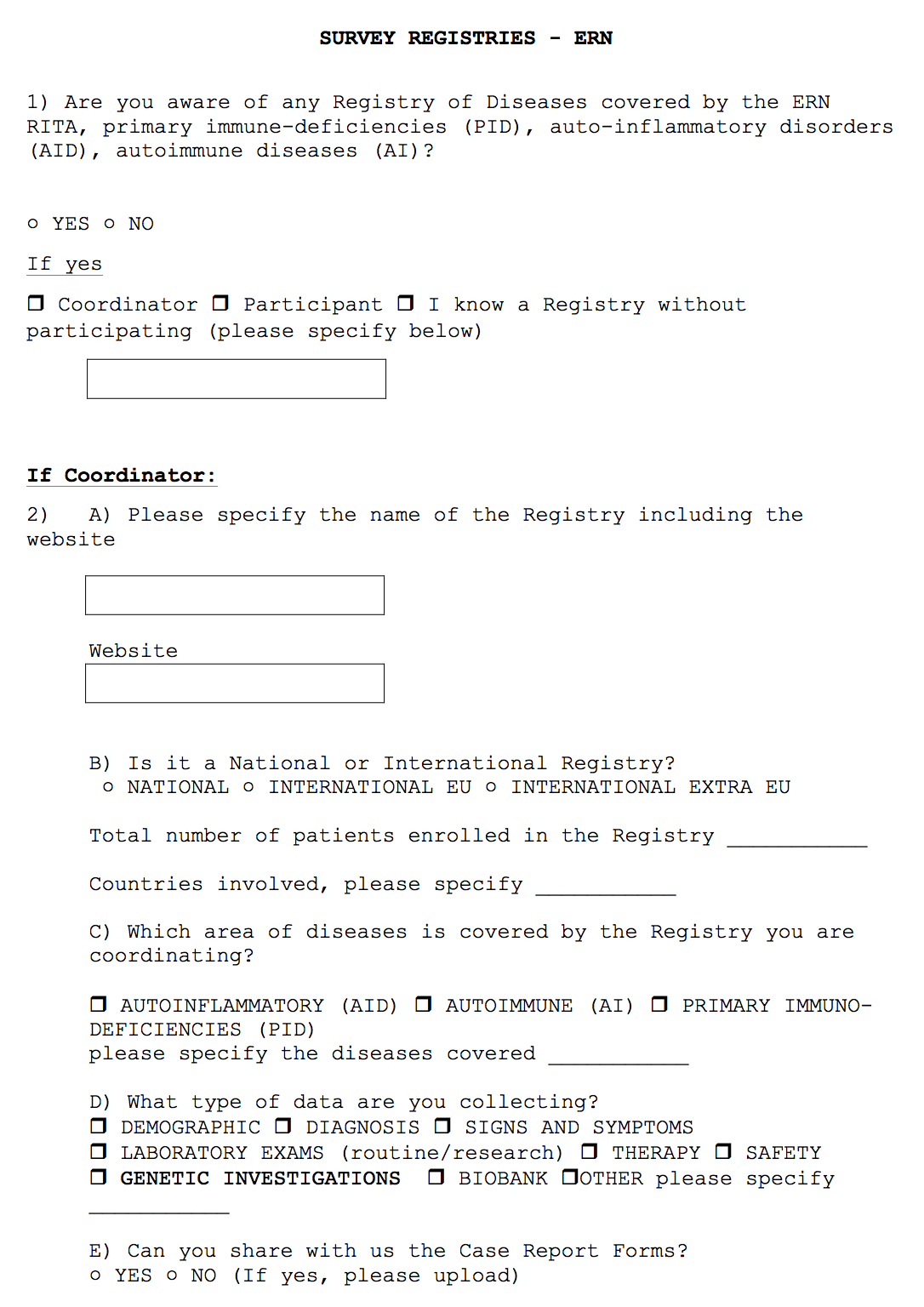


**
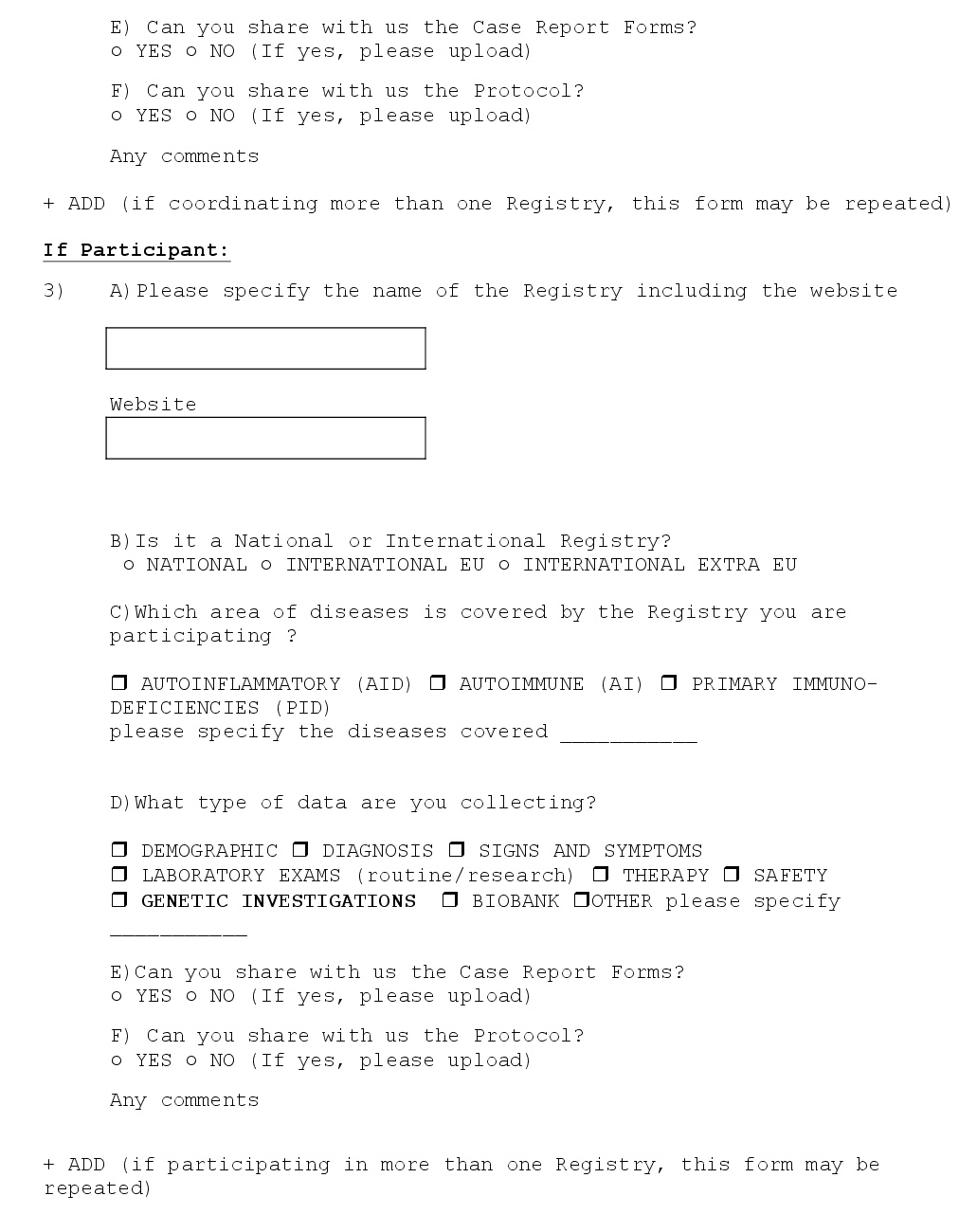
**

Supplement: Supplementary file 1 — Additional file 1. The registries survey sheet. [file 13023_2020_1308_MOESM1_ESM.docx]
